# Presumed Bietti crystalline dystrophy with optic nerve head drusen: a case report

**DOI:** 10.1186/s13256-022-03581-7

**Published:** 2022-11-02

**Authors:** Fatemeh Bazvand, Esmaeil Asadi Khameneh

**Affiliations:** Vitreoretina Department of Ophthalmology, Farabi Eye Hospital, Tehran University of Medical Sciences, Tehran, Iran

**Keywords:** Bietti crystalline dystrophy, Optic nerve head drusen, Retinal imaging

## Abstract

**Background:**

Bietti crystalline dystrophy is primarily a retinal dystrophy caused by a *CYP4V2* mutation and typically presents with crystalline retinal deposits in the posterior fundus.

**Case presentation:**

We present the case of an otherwise healthy 39-year-old Iranian woman with no family history of ocular disease who suffered with progressive vision loss that had started 2 years prior to presentation. Ocular examination revealed blurry optic nerve head margin and diffuse retinal crystalline deposit in both eyes. Spectral domain optical coherence tomography images showed retinal crystals, located mostly in outer retinal layers, with some areas of outer retinal tubulation and attenuation of outer retinal layers. Crystalline deposits were better visualized on near-infrared images as hyperreflective spots. Fundus autofluorescence images showed hyperautofluorescence areas on optic nerve head consistent with optic nerve head drusen and large hypoautofluorescence areas in posterior retina consistent with retinal pigment epithelium atrophy. Cystinosis was ruled out by blood testing.

**Conclusion:**

Bietti crystalline dystrophy may be associated with optic nerve head drusen.

## Introduction

Bietti crystalline dystrophy typically occurs in the second to fourth decades of life, with symptoms of reduced visual acuity with or without nyctalopia [1, 2]. Ocular examination revealed crystalline retinal deposits in the posterior fundus and crystalline corneal deposits near the limbus in nearly one-third of patients [2]. These crystals show hyperreflectivity on near-infrared (NIR) retinal images in spectral-domain optical coherence tomography (SD-OCT). These crystals can be seen in all retinal layers but are most prominent between retinal pigment epithelium and Bruch’s membrane [3, 4]. Bietti crystalline dystrophy is a typically autosomal recessive disorder caused by mutations in the *CYP4V2* gene that result in defective ocular fatty acid metabolism in retinal pigment epithelium and the formation of crystalline deposits [5–7]. In the advanced stages of the disease, retinal crystals may not be evident owing to the atrophy of retinal pigment epithelium [2]. In this study, we report on a case of Bietti crystalline dystrophy associated with optic nerve head drusen.

## Case report

A 39-year-old Iranian woman suffered from progressively reduced vision in both eyes that had started 2 years prior to presentation. Her parents were relatives, but there was no family history of ocular disease in the patient’s first- and second-degree family. The patient’s medical history and drug history were unremarkable. Blood testing and urine analysis were performed to rule out cystinosis and crystalluria. Written informed consent was obtained from the patient. A complete ocular examination—including optical coherence tomography (OCT), OCT angiography (OCT-A), infrared (IR) imaging, fundus autofluorescence (FAF) imaging, and electroretinography (ERG)—was performed.

### Ocular examination

**Fig. 1:**
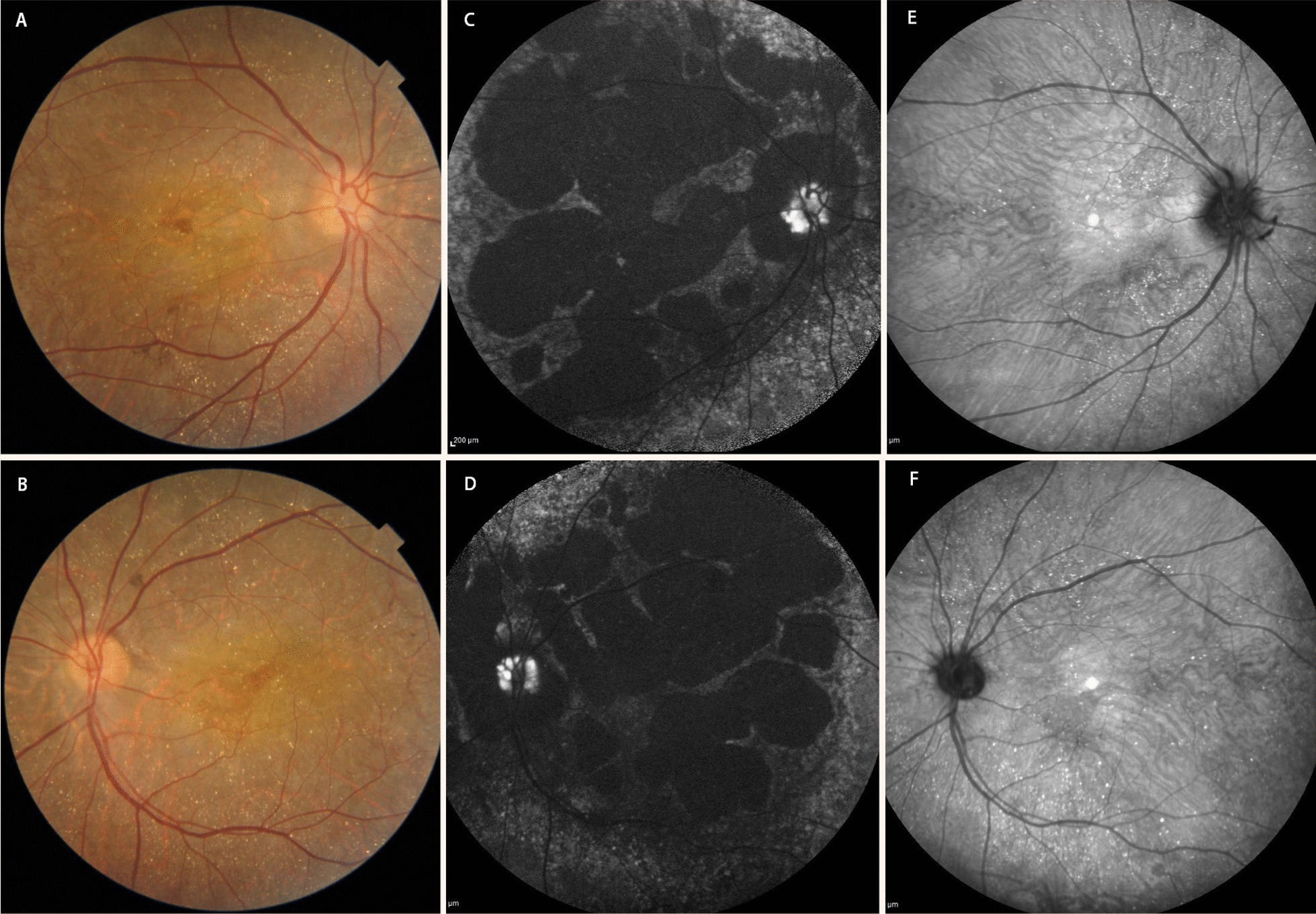
Fundus photograph of posterior pole in our patient showing crystals and blurry optic nerve head margin in right and left eye, respectively (**A** and **B**). Fundus autofluorescence images of right and left eye showing hyperautofluorescence areas on optic nerve head consistent with optic nerve head drusen in both eyes and hypoautofluorescence areas in posterior retina (**C** and **D**). Near-infrared images of the right and left eye, respectively, with better visualization of crystalline deposits (**E** and **F**)

The best-corrected Snellen visual acuity in both eyes was 7/10. Anterior segment examinations were normal. A posterior segment examination revealed a bilateral blurry optic nerve head margin, a bilateral diffuse crystalline deposit at the posterior pole through mid-periphery, and reduced foveal reflex (Fig. 1A, B).

### OCT findings

**Fig. 2:**
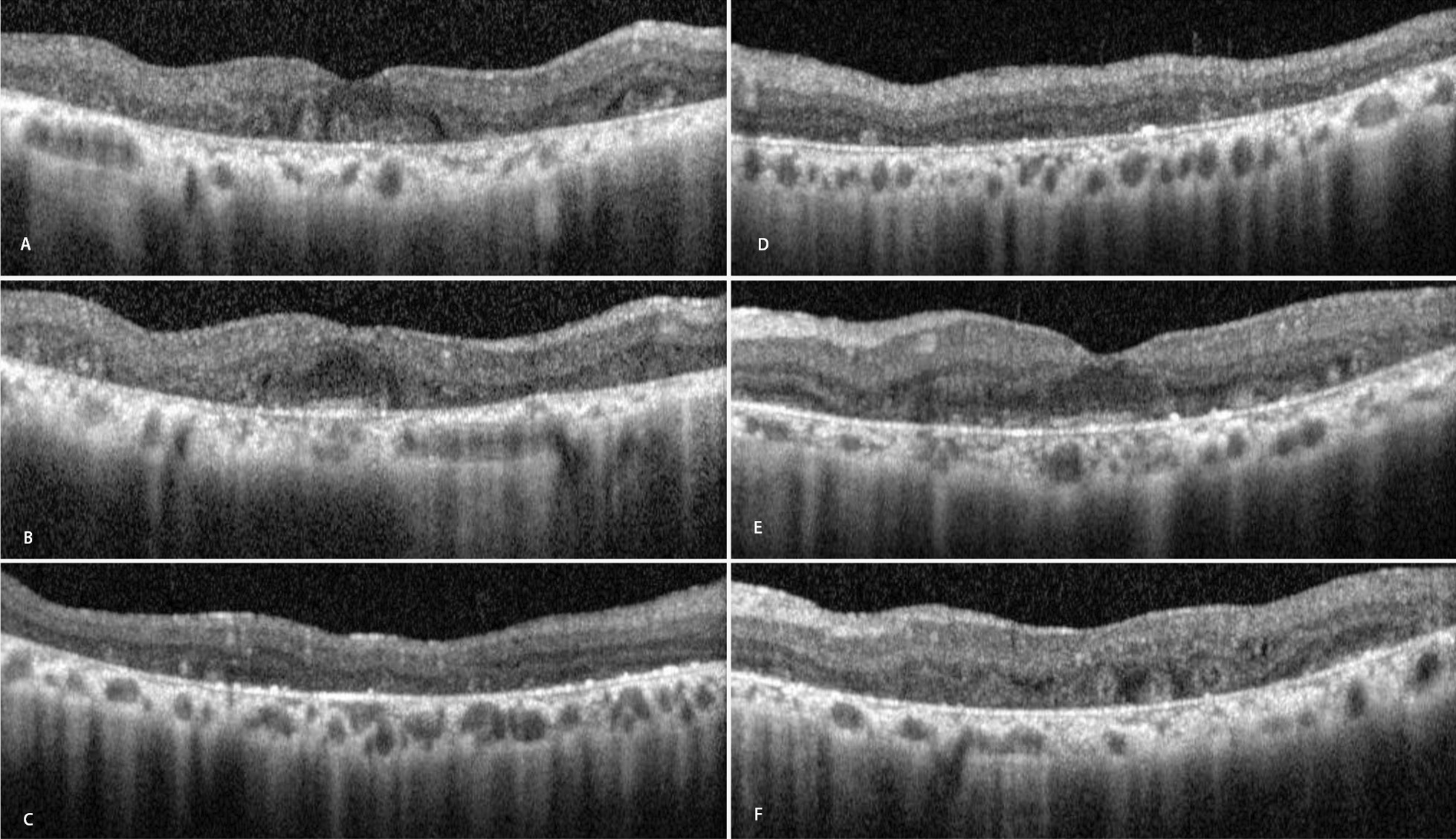
Spectral domain optical coherence tomography angiography of right (**A**–**C**) and left (**D**–**F**) eyes in macular area showing hyperreflective spots in outer retinal layers (better seen on **C**–**E**) along with outer retinal tubulation (better seen on **A**, **B**, and **F**), and choriocapillaris dropout that caused decreased backscattering

SD-OCT revealed patchy atrophy of the outer retina (Fig. 2B, E). OCT also revealed hyperreflective spots in all layers of the retina, especially within the retinal pigmented epithelium and Bruch’s membrane, along with outer retinal tubulation, outer retinal layers atrophic changes, and choriocapillaris dropout (Fig. 2A–F).

### IR and FAF findings

On NIR images, crystalline deposits were better visualized as hyperreflective spots throughout the posterior retina in both eyes (Fig. 1E, F). FAF images show hyperautofluorescence areas on the optic nerve head consistent with optic nerve head drusen in both eyes. Large patchy hypoautofluorescence areas were seen in the posterior retina of both eyes, which is consistent with retinal pigment epithelium atrophic changes that were seen in SD-OCT images (Fig. 1C, D).

### OCT-A findings

**Fig. 3:**
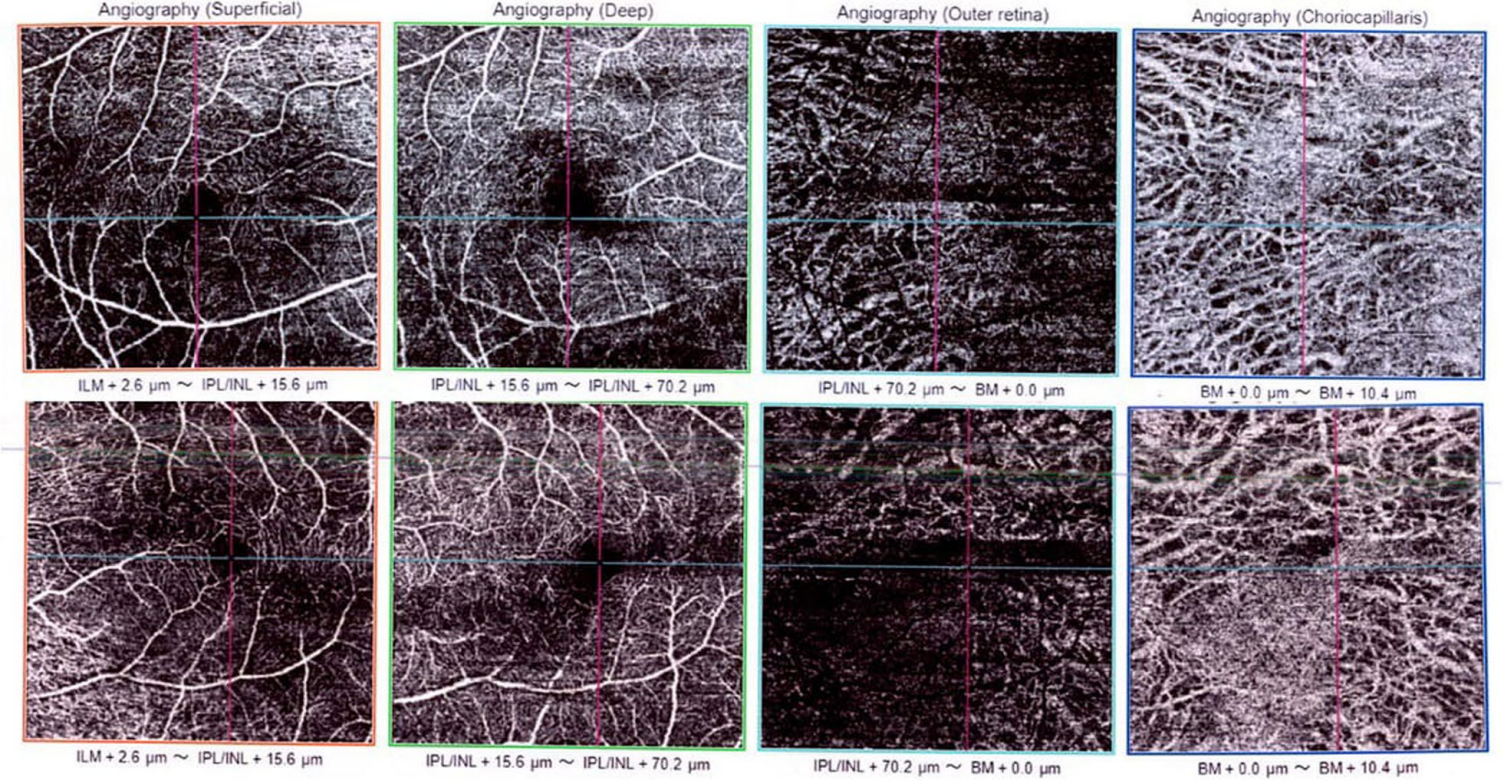
Optical coherence tomography angiography of right (top row) and left (bottom row) eyes of the patient in four levels (superficial and deep capillary plexuses, outer retina, and choriocapillaris level, respectively, from left to right)

The foveal avascular zone was irregular, especially in the right eye in superficial and deep capillary plexuses (Fig. 3). Large choroidal vessels of the Henle layer were seen on the choriocapillaris slab of OCT-A images owing to choriocapillaris atrophy (Fig. 3).

### ERG findings

**Fig. 4:**
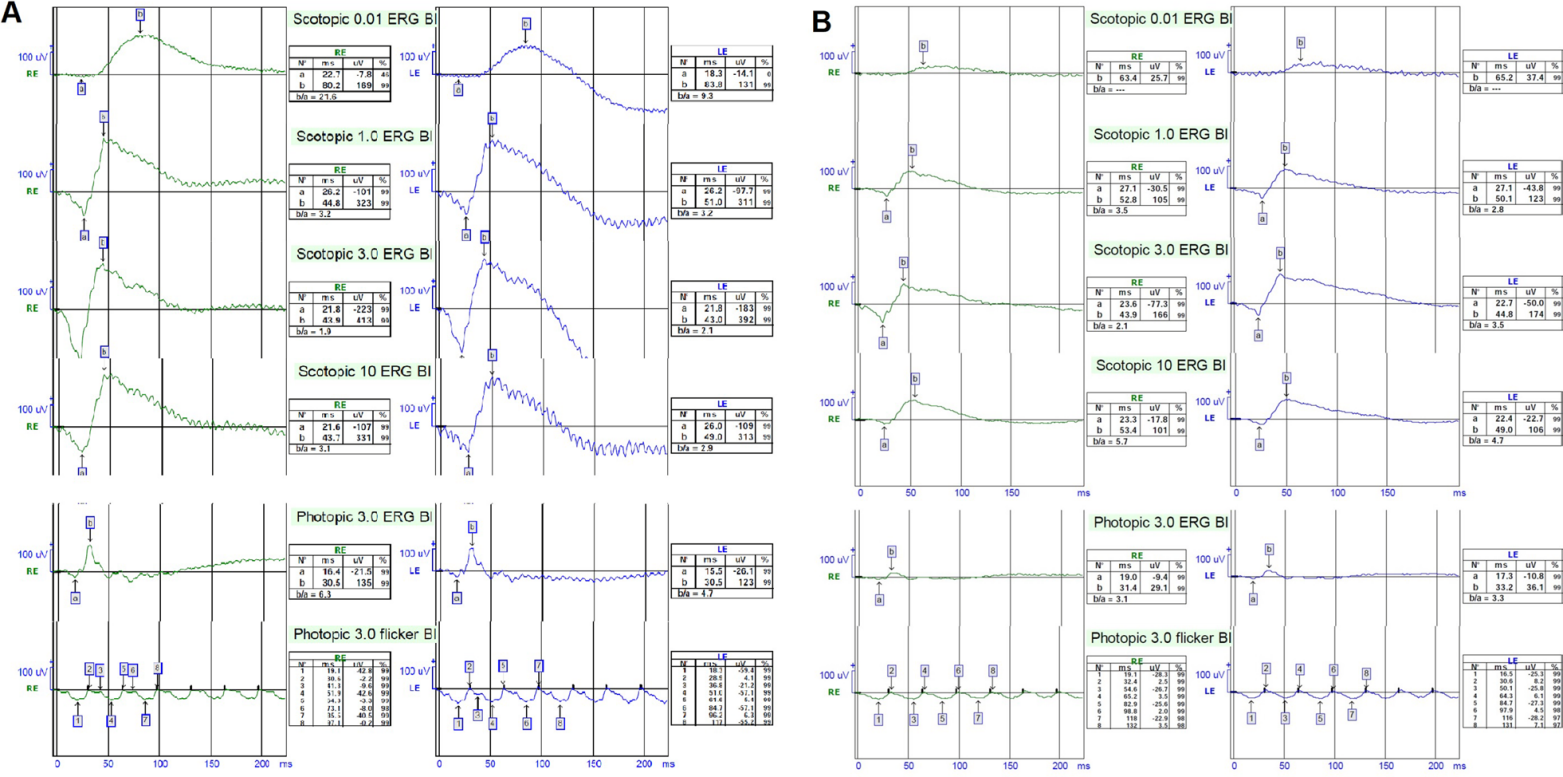
Normal electroretinogram (**A**). Electroretinogram of our patient (**B**) showing a moderate reduction of both a and b waves in photopic and scotopic responses

ERG showed moderate to severe reductions in both photopic and scotopic responses (Fig. 4).

The most probable diagnosis according to the clinical examination and multimodal imaging was Bietti crystalline dystrophy with bilateral optic nerve head drusen. However, the confirmation of the diagnosis requires genetic testing. Genetic testing of *CYP4V2* mutation was not performed for the patient owing to economic restraints.

## Discussion

Bietti crystalline dystrophy is primarily a retinal dystrophy caused by a *CYP4V2* mutation and is presumed to affect fatty-acid omega-hydroxylase activity in retinal pigmented epithelium and accumulation of yellow-white crystalline-like deposits [1, 2, 8]. These crystals can also be found in the peripheral cornea near the limbus [2]. This disease is frequently inherited as an autosomal recessive pattern, but an autosomal dominant pattern has also been seen within family members who have the disease [1, 5, 7]. These patients typically become symptomatic during the third decade of life via bilateral slowly progressing vision loss, nyctalopia, and visual field constriction. The rapid deterioration of vision in these patients should increase suspicion of the development of cystoid macular edema, choroidal neovascularization, and macular hole formation [9, 10]. In the early stage of the disease, retinal crystals are prominently found in the posterior retina with minimal retinal pigment epithelium atrophic changes in the macula. With progression of the disease, the retinal pigment epithelium atrophic changes in the macular area worsen, and the presence of retinal crystals decreases in the posterior pole while increasing outside the vascular arcades and peripheral retina. In the advanced stage of the disease, extensive retinal pigment epithelium atrophic changes are seen throughout the fundus, and retinal crystals may not be evident [2, 10]. These crystals could be found in all layers of the retina, but, as with our patient, they are more numerous between retinal pigment epithelium and Bruch’s membrane [4]. These crystals are better visualized on NIR fundus images, and they are hyperreflective on SD-OCT images [4]. SD-OCT images also show outer retinal tubulation in many patients with Bietti crystalline dystrophy, even in the early stages of the disease. Other posterior segment findings in these patients are choriocapillaris atrophy, choroidal sclerosis, and the disruption of outer retinal hyperreflective bands [2, 4, 11]. Outer retinal tubulation and the disruption of the ellipsoid band on SD-OCT images (both of which were seen in our case) predict poor visual outcomes in these patients [11]. The sensitivity and specificity of NIR images for detecting retinal crystals and sensitivity of SD-OCT for detecting outer retinal tubulation in these patients are high, but outer retinal tubulation is not specific for this disease [3, 4, 9, 11]. Retinal pigment epithelium atrophic changes are better visualized on FAF images as areas of reduced autofluorescence due to the atrophy of retinal pigment epithelium. Fluorescein angiography in the early stages of the disease typically shows window defects due to the atrophy of outer retinal layers and retinal pigment epithelium; however, as the disease progresses, choriocapillaris atrophy causes early hypofluorescence and late hyperfluorescence due to the staining of underlying sclera [4, 12]. Fluorescein angiography and OCT-A could help establish diagnoses of choroidal neovascularization as a complication of the disease in a proper clinical context. Full-field ERG studies typically show diminished photopic and scotopic responses [2, 3]. The clinical examination and multimodal imaging of our patient were typical for Bietti crystalline dystrophy. The patient’s medical and drug histories were negative, and blood tests for cystinosis and urine analysis yielded normal results. Bietti crystalline dystrophy was diagnosed without applying genetic testing to confirm the diagnosis.

Optic nerve head drusen is a clinical condition that is usually asymptomatic and found incidentally but may be associated with retinal hemorrhage and visual field defects [13]. Optic nerve head drusen mainly consists of calcified hyaline bodies and occurs in up to 2% of the general population [13]. Optic nerve head drusen typically appears as hyperreflectivity on SD-OCT images and hyperautofluorescence on FAF images, as seen in our patient.

## Conclusion

To the best of our knowledge, this is the first report on the association between optic nerve head drusen and Bietti crystalline dystrophy.

## Data Availability

Not applicable

## Abbreviations

NIR: Near-infrared
SD-OCT: Spectral-domain optical coherence tomography
OCT: Optical coherence tomography
OCT-A: Optical coherence tomography angiography
IR: Infrared
FAF: Fundus autofluorescence
ERG: Electroretinography
